# Confinement suppresses instabilities in particle-laden droplets

**DOI:** 10.1038/s41598-017-08126-3

**Published:** 2017-08-09

**Authors:** Lalit Bansal, Saptarshi Basu, Suman Chakraborty

**Affiliations:** 1 Department of Mechanical Engineering, Indian Institute of Science, Bangalore, 560012 India; 2 Department of Mechanical Engineering, Indian Institute of Technology Kharagpur, Kharagpur, 721302 India

## Abstract

Tiny concentrations of suspended particles may alter the behavior of an evaporating droplet remarkably, leading to partially viscous and partially elastic dynamical characteristics. This, in turn, may lead to some striking mechanical instabilities, such as buckling and rupture. Here, we report certain non-trivial implications of the consequent morpho-dynamics (macro to nano scales), when such an evaporating droplet is encapsulated in a confined environment. Compared to unconfined scenario, we report non-intuitive suppression of rupturing beyond a critical confinement. We attribute this to confinement-induced dramatic alteration in the evaporating flux, leading to distinctive spatio-temporal characteristics of the internal flow leading to preferential particle transport and subsequent morphological transitions. We present a regime map quantifying buckling-non buckling pathways. These results may turn out to be of profound importance towards achieving desired morphological features of a colloidal droplet, by aptly tuning the confinement space, initial particle concentration, as well as the initial droplet volume.

## Introduction

Dynamics of drying suspensions find intriguing applications in diverse fields, ranging from spray drying^1–4^ to forensic investigations^5, 6^. In spray drying, fine particles are produced by rapid evaporation of aerosols, which may act as a basis for producing large quantities of food stuff ^7, 8^, pharmaceuticals^9^, polymers, and detergents. On an entirely different perspective, when a crime results in bloodshed, the drying droplets of blood left behind may act as a source of evidence for the investigations. Patterns of drying help the analysts on assessing the length of the assault, its various stages, and pinpointing probable crime scene contamination.

Drying suspensions often exhibit a blend of viscous and elastic behavior^10–13^. In addition, their kinetics of evaporation may drive the suspensions far from equilibrium. However, unearthing a clear picture on the underlying interplay between forces, structure, kinetics and confinement appears to remain as an outstanding question in interdisciplinary physics. In particular, exactly how confinement influences the onset of rupture and buckling, as well as the associated morpho-dynamics, remains to be elucidated. A deeper understanding requires careful investigation of drying suspensions subjected to tunable and well defined confined fluidic environments.

Here, we describe the confinement-induced non-trivial alterations in the evaporating dynamics of freely suspended droplets of colloidal suspensions. We show that when evaporating in a channel of length (*L*_*c*_) below a threshold limit, a droplet behaves as if it is unconfined and undergoes buckling as well as rupturing. However, as *L*_*c*_ is increased, rupturing is suppressed. We also unveil a critical *L*_*c*_, beyond which even buckling is eliminated, resulting in dome shaped precipitate, which may alternatively be realized by reducing the initial particle concentration (φ_o_) that would result in flatter precipitates. We bring out a regime diagram depicting certain universal features of the coupling among the confinement, evaporation, internal flow evolution, particle accumulation, structure formation and the eventual morphology (macro-nano scales).

## Results

### Evaporation and self-assembly of particles

**Figure 1:**
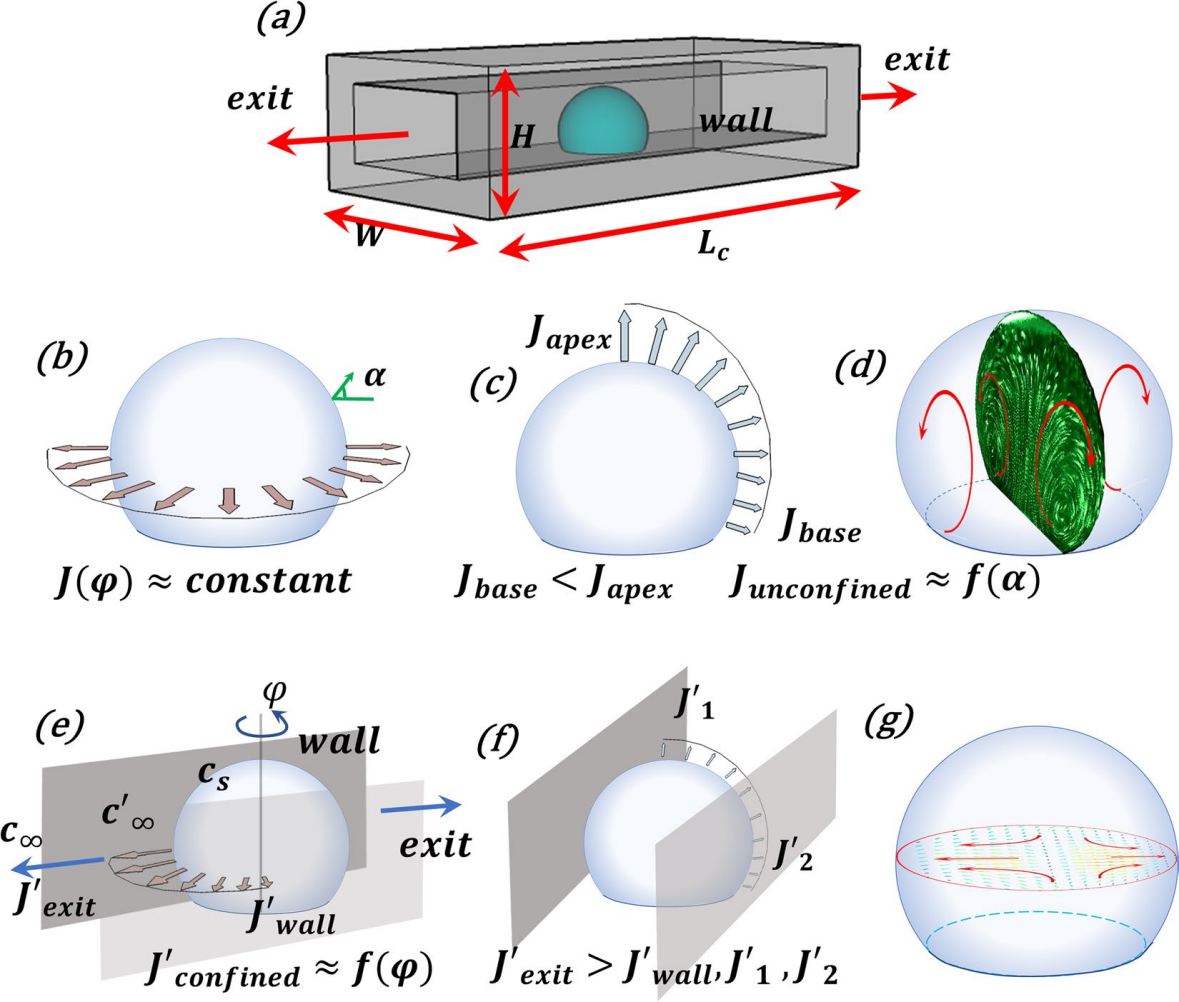
Overview of the experimental conditions. (**a**) Schematic showing the confined droplet. Variation in evaporation flux and flow patterns for unconfined (**b**–**d**) and confined (**e**–**g**) droplets. For unconfined droplets, evaporation is uniform in azimuthal direction (**b**) rather than polar direction (**c**) resulting in recirculating toroidal flow (**d**). For confined droplets, evaporation is comparatively more non-uniform in azimuthal direction (**e**) than polar (**f**). This leads to a directional flow from wall to open side (**g**). Arrows around the droplet periphery denote the evaporation flux. Channel top plate is not shown for simplicity. Here, ${c}_{\infty }^{^{\prime} }$: increased vapor concentration in the channel due to entrapment, *c*_∞_ and *c*_*s*_: ambient and saturated vapor concentrations respectively.

**Figure 2:**
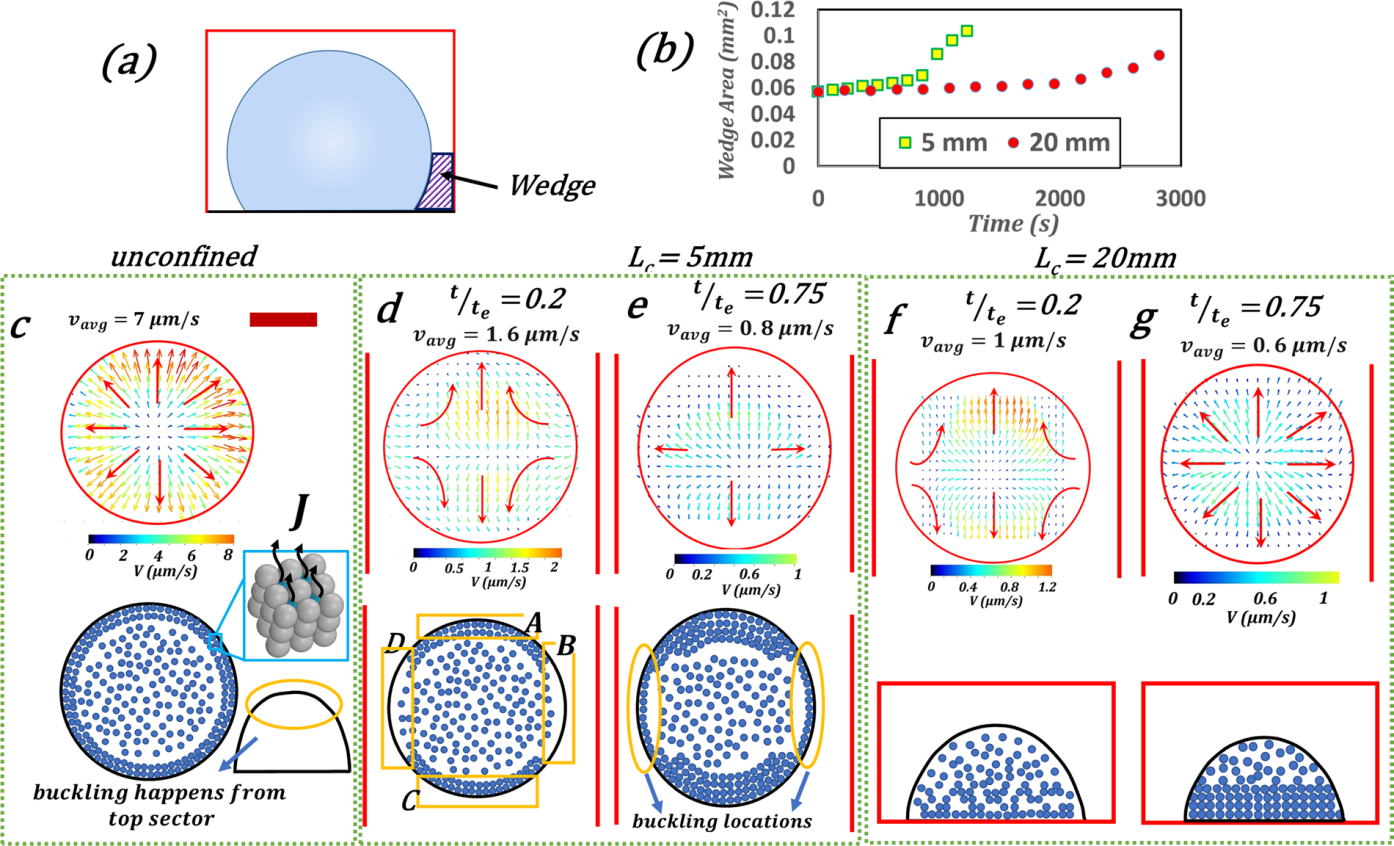
Flow field and particle deposition. (**a**) Representative image of the wedge. (**b**) Temporal variation in wedge area. Internal velocity field of the droplet (measured at $h/{h}_{i}=0.5$ where *h*_*i*_: initial droplet height) and corresponding schematic showing particle aggregation (second row) for (**c**) unconfined case where the radially outward flow results in azimuthally uniform particle deposition (**d**,**e**) *L*_*c*_ = 5 mm; directional flow leads to thicker shell on the open sides; buckling occurs from the wall (thinner) sides (**f**,**g**) *L*_*c*_ = 20 mm; dome shaped structure is obtained due to low evaporation flux. For all cases, *φ*_*o*_ = 40 *wt*.%. Red arrows depict the flow directions. Channel walls are depicted by red lines. Inset in (**c**) shows the evaporation process from nano-menisci. t_e_: total time for evaporation. Scale bar equals 500 µm.

For an unconfined droplet on a hydrophobic substrate, evaporation is uniform in the azimuthal direction (Fig. 1b) but not in the polar direction^12^ (Fig. 1c). From energy conservation, one can equate the conduction flux with the evaporation flux and write:$\,{k}_{c}\frac{{\rm{\Delta }}T}{R}\sim J{h}_{fg}$ or Δ*T* ∝ *J* ∝ (*c*_*s*_ − *c*_∞_). Here Δ*T* (=*T*_*c*_ − *T*_*s*_): the temperature difference between the droplet center (*T*_*c*_) and surface (*T*_*s*_), *k*_*c*_: thermal conductivity, *R*: droplet local radius, evaporative flux: *J* ∝ (*c*_*s*_ − *c*_∞_); *c*_∞_ and *c*_*s*_ being the ambient and saturated vapor concentration (at 25 °C) respectively. Moreover, suppression of evaporation near the three-phase contact line results in *J*_*apex*_ > *J*_*base*_ (Fig. 1c) $(\frac{{J}_{apex}}{{J}_{base}}\approx 3)$. Thus, Δ*T*_*apex*_ > Δ*T*_*base*_ or *T*_*apex*_ < *T*_*base*_ (Supplementary Fig. S2) which leads to *ρ*_*base*_ < *ρ*_*apex*_ where *ρ*: nanofluid density. This creates a toroidal recirculating flow (buoyancy controlled) inside the droplet^14, 15^ (Fig. 1d) whose average velocity, *v*_*uc*_ ∝ Δ*J* (=*J*_*apex*_ − *J*_*base*_) (subscript ‘uc’: unconfined). Thus, internal flow is a linear function of the maximum difference in spatial evaporation flux rather than its absolute value. The flow therefore causes preferential particle aggregation (Supplementary Fig. S4), leading to the formation of a spatially inhomogeneous nano-porous elastic shell. The shell undergoes buckling at the weakest location (lowest shell thickness) located in the top sector of the droplet (Fig. 2c). Furthermore, this buckled shell generally ruptures, creating a cavity inside the droplet^12^.

**Figure 3:**
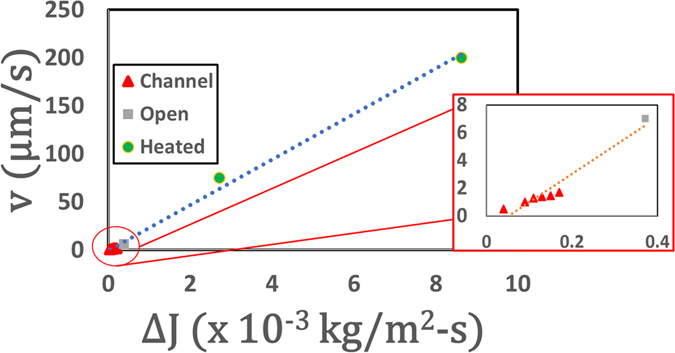
Velocity as a function of spatial variation in evaporation rate. Inset shows the magnified view of the channel data. Heated data is taken from [ref. 15]. For open and heated cases, J variation is determined using the equation $J=-D(\frac{\partial c}{\partial r}\,{\cos }\,\alpha +\frac{\partial c}{\partial z}\,{\sin }\,\alpha )$ ^18^ where D: diffusion coefficient, c: vapor concentration and α: as shown in the Fig. 1b. For confined droplet, *J*′ variation is in the azimuthal direction, i.e., ${\rm{\Delta }}{J}_{c}={J}_{exit}^{^{\prime} }-{J}_{wall}^{^{\prime} }\approx {J}_{exit}^{^{\prime} }\approx {J}_{channel}^{^{\prime} }$.

For a droplet evaporating in a confined fluidic environment (Fig. 1a), vapor entrapment in the wedge formed (Fig. 2a) by the droplet interface, channel wall and its base suppresses the global evaporation flux (*J*′)^16, 17^. This results in an increase in local vapor concentration $({c}_{\infty }^{^{\prime} }=f({L}_{c}))$ (Fig. 1e). However, unlike for the unconfined case, here $({J}_{apex}^{^{\prime} }-{J}_{base}^{^{\prime} })\approx 0$ and ${\rm{\Delta }}{J}_{c}={J}_{A-C\,(exit)}^{^{\prime} }-{J}_{B-D\,(wall)}^{^{\prime} }\,$(Fig. 2d); (subscript ‘c’: confined), due to spatial variation in the azimuthal direction (Fig. 1e) rather than in the polar direction (Fig. 1f). Vapour entrapment on wall side allows us to neglect ${J}_{wall}^{^{\prime} }\,$ which gives ${\rm{\Delta }}{J}_{c} \sim {J}_{exit}^{^{\prime} }$. The azimuthal variation in Δ*J*_*c*_ and lower absolute value of *J*′ has a two-fold effect on the flow field. First, even slight confinement incurs a drastic reduction in Δ*J* (*L*_*c*_ = 5 *mm*) which induces a large reduction in velocity (~80% for this case). In addition, the velocity further reduces with an increase in *L*_*c*_ since ${v}_{c}\propto {\rm{\Delta }}{J}_{c}\sim {J}_{A-C\,(exit)}^{^{\prime} }\propto 1/{L}_{c}$(Figs 3 and S3). However, it has been observed from our preliminary studies that increasing the height or width relaxes the confinement effect thus accelerating the evaporation dynamics (for example, $\frac{{t}_{{\rm{e}},4mm,width}}{{t}_{{\rm{e}},2mm,width}}=0.66$ for 1.3 mm height where *t*_e_ is the total evaporation time).

**Figure 4:**
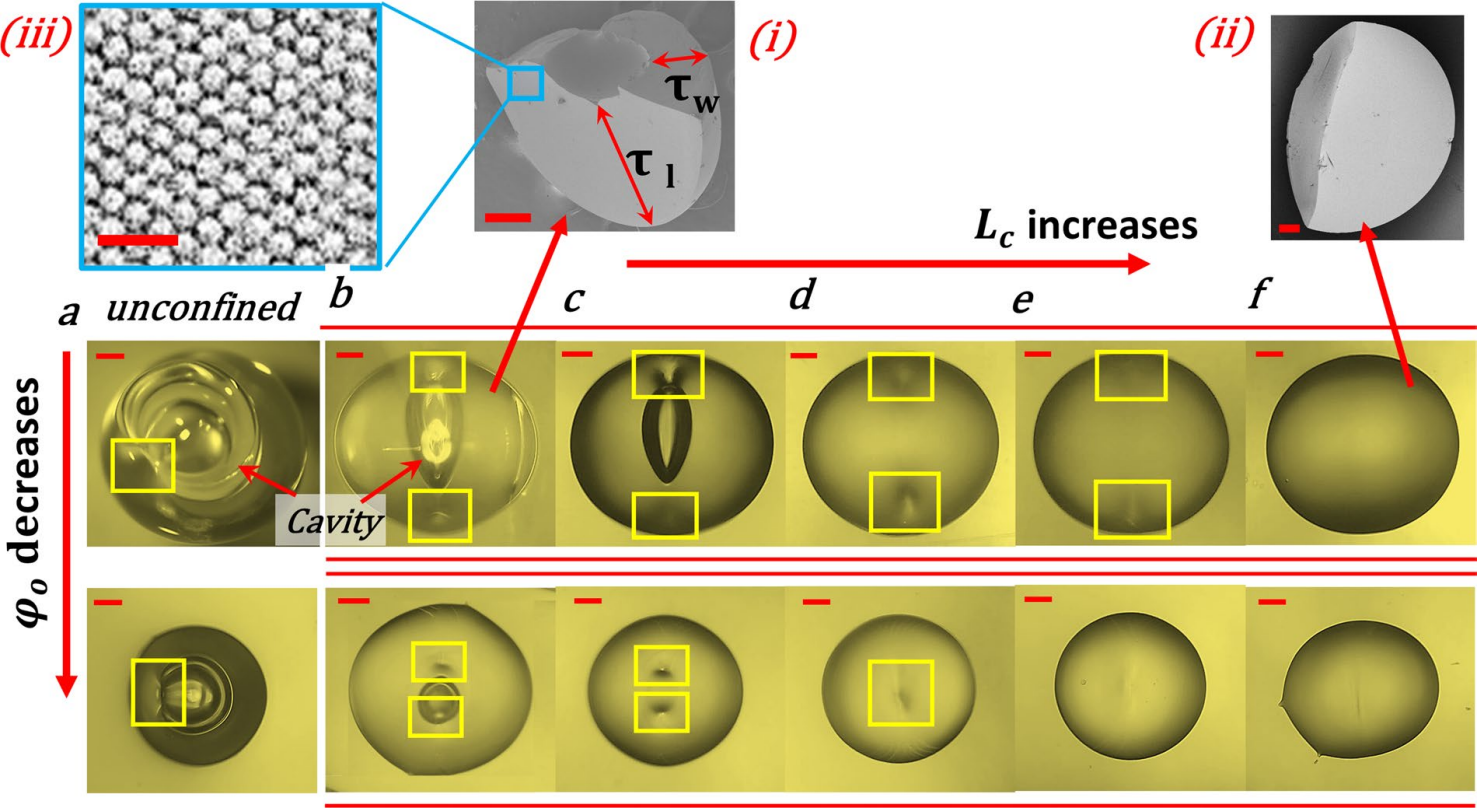
Decrease in buckling tendency. Droplet morphology showing droplet final structures (top view) for (**a**) unconfined case (**b**–**f**) confined cases with *L*_*c*_ = (**b**) 5 mm (**c**) 7 mm (**d**) 10 mm (**e**) 15 mm (**f**) 20 mm. Images shown in second row correspond to same confinements with *φ*_*o*_ = 15 *wt*.% whereas first row *φ*_*o*_ = 40 *wt*.%. (**a**) Buckling and subsequent rupturing is observed in top sector of the droplet irrespective of concentration. (**b**–**e**) For *φ*_*o*_ = 40 *wt*.% buckling locations are shifted to the wall sides. (**b**,**c**) Buckling is followed by rupturing, (**d**–**e**) only buckling is observed (**f**) buckling is completely suppressed with a dome shaped structure. For confined cases with 15wt.%, buckling locations shift back to the top sector. Inset (i) and (ii)-SEM micrographs of the final precipitates for channel length *L*_*c*_ = 5 mm and 20 mm respectively. τ_l_ and τ_w_ correspond to the aggregate thickness on the exposed portion and wall side respectively. (iii) SEM micrograph showing the final particle arrangement. Yellow boxes denote the buckling locations and large red horizontal lines represent the channel walls. Scale bar equals 200 µm. Scale bar in the inset (i) and (ii) equals 100 µm while in (iii) equals 60 nm.

Secondly, non-uniform evaporation pattern in the azimuthal direction changes the flow field from recirculating toroidal (unconfined) to directional (towards the open side of the channel; Figs 1g and 2d). As a result, there is azimuthally varying particle deposition, the maximum being at the droplet surface closer to the open side (Fig. 2e). Subsequently, buckling location is shifted from the top sector (Fig. 4a) towards the droplet surface near the channel walls (Fig. 4b–e). Furthermore, for short channels, buckling is followed by rupturing (Fig. 4b,c). However, with an increase in *L*_*c*_, buckling tendency subsequently is arrested, with a complete suppression beyond a critical length (Fig. 4f and inset 4 (ii)).

How can we explain the physics of the phenomena depicted as above? Towards addressing this, we first note that pure water droplet (confined or unconfined) exhibits three modes of evaporation namely, constant contact radius (CCR), constant contact angle (CCA) and mixed. On the contrary, for colloidal droplets in a confined environment, the initial evaporation proceeds in CCR mode (Supplementary Fig. S1). As evaporation progresses, the contact line gets prematurely pinned (due to particle deposition). However, for confined droplets, evaporation modes are different along the channel length and width. Preferential evaporation from regions A-C pins the three-phase contact line restricting the final CA to *θ*_*L*_ ~ 100 ± 2° for L_c_ = 5 mm and *θ*_*L*_ ~ 90 ± 2° for L_c_ = 20 mm (*θ*_*L*_: CA viewed from side wall). The variation in final dynamic CA with L_c_ is due to the corresponding variations in Δ*J*_*c*_. Preferential pinning of the contact line also transforms the droplet from spheroidal to ellipsoidal.

For a particular L_c_, the initial particle concentration also affects the evaporation dynamics. At low φ_o_, there is a decrease in particle deposition rate (explained later), thereby delaying the contact line pinning in regions A-C as compared to higher PLR. This enhances interfacial slip in that region, resulting in spheroidal droplet shape (Fig. 4).

In contrast, dynamic CAs in wall regions B–D continue to decay due to negligible particle deposition. However, when *θ*_*w*_ decays below 90° (*θ*_*w*_: CA viewed from the open side), wedge area increases (Fig. 2b) which relaxes the wall effect thereby initiating contact line slip. This creates a radially outward flow profile. Even though evaporation from regions B-D are not yet completely free from the wall effect (Fig. 2e) $(\frac{{v}_{c(A-C)}}{{v}_{c(B-D)}}\approx 3)$, the rate of particle deposition gets enhanced with time (See Supplementary Movie M1) locking the final *CA* ~ 70°.

We focus next on the flow driven particle deposition and subsequent structure (like shell) formation dynamics. For confined cases, apart from *φ*_*o*_, particle deposition rate and hence shell formation is a function of L_c_ (Fig. 4). Let us first consider the channel configurations leading to shell formation (*L*_*c*_ = 5 *mm*–15 *mm* and *L*_*c*_ = 5 *mm*–10 mm for *φ*_*o*_ = 40 *wt*.% and 15 *wt*.% respectively (Fig. 4)). There are significant differences in the amount of particle deposition along the channel length (*τ*_*l*_) and width (*τ*_*w*_) in regions A-C and B-D respectively (Fig. 4 inset i & ii) due to azimuthal asymmetry (Supplementary Movie M1). From particle mass conservation, one can show that for any L_c_, (See Supplementary Fig. S5 for details)

1 $$\tau =\frac{{v}_{c}{\phi }_{o}{\rm{\Delta }}t}{{\phi }_{p}}$$

where *φ*_*p*_: final particle packing fraction in the precipitate. For *τ*_l_, Δ*t*: time elapsed between pinning of the contact radius and the total evaporation time (*t*_*e*_) in region A-C. On the other hand, for *τ*_*w*_, Δ*t* is the time difference between the instant at which the flow returns to the radially outward pattern and buckling onset. Assuming loose random packing (Fig. 4 inset iii) due to slow evaporation inside the channel, we have *φ*_*p*_ = 0.56^19^. The shell thickness, *τ*_l_ (Eqn. 1) is found to be in agreement with the experimental values (from SEM images (Supplementary Fig. S6) (*τ*_*l*,*exp*_ ~0.6 − 08 mm for L_*c*_ = 5 mm–15 mm), i.e. $\frac{{\tau }_{l,theo}}{{\tau }_{l,exp}}\approx 0.9\pm 0.02$ irrespective of L_c_. On the contrary, *τ*_*w*_ is estimated to be 10 ± 2 *μm*. This is of the same order as that obtained by Miglani *et al*.^20^ (~15 ± 7 *μm*) for levitated droplets and Kim *et al*.^21^ (buckled layer thickness ~4–5 *µm*) for polystyrene colloids. Thus, the points of minimum shell thickness are located in regions B-D (${\tau }_{l,exp}\gg {\tau }_{w}$).

Furthermore, shell only forms for *φ*_*o*_ > *φ*_*m*_ where *φ*_*m*_ is the minimum PLR that varies with L_c_. There is an increase in *φ*_*m*_ from 5wt.% for unconfined case to 10wt.% for confined case (L_c_ = 5 mm) (decay in *J*). For *φ*_*o*_ < *φ*_*m*_, there are insufficient particles in the dispersion to sustain the shell formation leading to thin disc shaped structures.

Another interesting thing to note from Fig. 4 is that as the PLR decreases, there is a subsequent shift in the location of minimum shell thickness back to the top sector. Enhanced contact radius slip (explained before) accompanied by a larger decrease in the droplet height (as compared to high *φ*_*o*_) $(\frac{{R}_{c,5m{m}_{40 \% }}}{{R}_{c,5m{m}_{15 \% }}}\approx 1.35\,and\frac{{h}_{5m{m}_{40 \% }}}{{h}_{5m{m}_{15 \% }}}\approx 2)$ results in a comparatively uniform particle deposition at the contact line in azimuthal direction. The combined effect of the aforementioned conditions shifts the point of minimum shell thickness towards the top sector. This effect is aggravated as *φ*_*o*_ is decreased further.

### Buckling and rupturing

**Figure 5:**
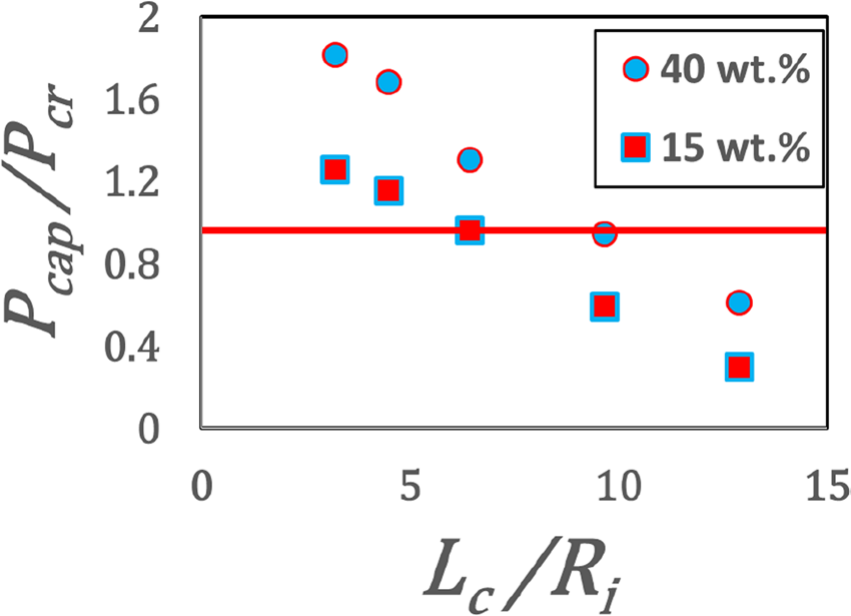
Buckling Criteria. Ratio of capillary pressure and critical pressure required for buckling for various normalized channel lengths (*L*_*c*_). *R*_*i*_ ≈ 1.52 is the initial droplet meridional radius. Red line marks the transition from buckling (>1) to non-buckling regime (<1).

Now, only for the cases with a distinct shell, water evaporation through the nano-menisci formed between the particles exerts a capillary force on the elastic shell. When this capillary pressure (*P*_*cap*_) overcomes the critical pressure (*P*_*cr*_) i.e. when $\frac{{P}_{cap}}{{P}_{cr}}\ge 1$, the shell buckles from the weakest spot (in regions B-D) forming a primary cavity. Buckling spot follows the loci of minimum shell thickness. From Darcy’s law, one can show ${P}_{cap}=\frac{\mu J{\tau }_{w}}{k}$ where *μ*: dynamic viscosity of the solvent, and *k*: permeability obtained from Carman-Kozeny equation; thin shell buckling theory dictates ${P}_{cr}=\frac{10{\tau }^{2}}{{R}_{B}^{2}}$ [ref. 22]. From Fig. 5, it is interesting to observe that as the ratio $\frac{{P}_{cap}}{{P}_{cr}}$ approaches 1, there is a corresponding decrease in the size of the buckled cavity. For $\frac{{P}_{cap}}{{P}_{cr}} < 1$, there is no buckling (Fig. 5).

Additionally, as evaporation continues, these buckled cavities may rupture if the stretching force during the growth of primary cavity overcomes the van der Waals force of attraction between the particles forming a daughter cavity inside the droplet. Thus, equating the two forces, the limiting value of strain $({{\epsilon }}_{lim})$ in the shell at the point of rupturing can be determined. Stress due to stretching is given by ${\sigma }_{s}=E\cdot {{\epsilon }}_{lim}$ where *E*: Young’s Modulus ~10^7^ Pa [ref. 13]; van der Waals stress between two spherical particles^23^ is given by ${\sigma }_{v}=\frac{A}{12{z}^{2}\cdot a}$ where *A*: Hamaker constant = 0.83 × 10^−20^ *J*, *z*: particle separation distance ~0.2 *nm* [ref. 13] and *a*: nanoparticle radius ~11 *nm*. Hence, from *σ*_*s*_ = *σ*_*v*_, we have ${{\epsilon }}_{lim}=0.14$. As an e.g. for *φ*_*o*_ = 40 *wt*%, *L*_*c*_ = 5 *mm*, ${{\epsilon }}_{exp}(0.16) > {{\epsilon }}_{lim}$ while for *L*_*c*_ = 10 *nm*, ${{\epsilon }}_{exp}( \sim 0.1) < {{\epsilon }}_{lim}$. Thus, rupturing is observed in the former case and not in the latter. This means that any buckled shell will rupture if the deformation amplitude (δ) exceeds a critical value defined by ${{\epsilon }}_{lim}$. During stretching^24^, $\delta \sim k^{\prime} \mu {R}_{c}^{2}{J}^{2}{E}^{-1}$; *k*′: constant. Thus, for constant *φ*_*o*_ and R_c_, we have, *δ* ∝ *J* ^2^ or ${\epsilon }\propto {J}^{2}\propto 1/{L}_{c}^{2}$. Hence, as confinement increases there is a decrease in rupturing tendency. In confined droplets, even though there are two buckling locations^12^, only one of them ruptures.

**Figure 6:**
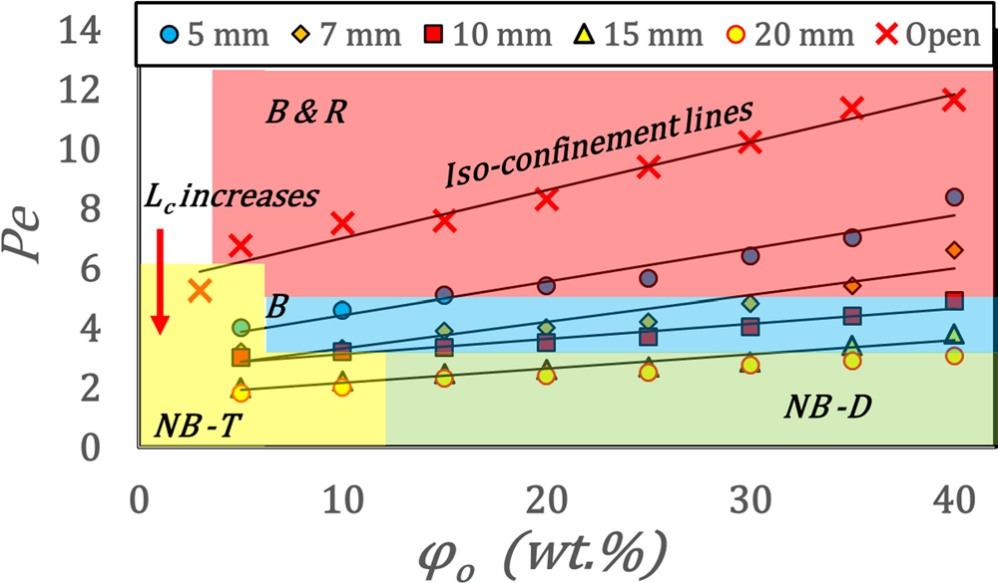
Regime map showing various final droplet morphologies for confined (different L_c_) and unconfined cases at different *φ*_*o*_. Notations used are B & R–Buckling and rupturing, B–Only buckling, NB-D–no buckling with dome shape and NB-T–No buckling with disc shape.

It is clear that shell formation is a necessary condition for buckling. However, as confinement is increased, the shell formation is gradually inhibited. As such, no distinct shell is observed for L_c_ = 20 mm irrespective of PLR (Fig. 4). Furthermore, we can categorize four distinct morpho-dynamic transition zones namely buckling (B), buckling-rupturing (B&R) and non-buckling - thin disc (NB-T) and dome shape (NB-D) in final precipitate morphology for different confinements and PLRs (Fig. 6).

These transitions can be better explained universally by resorting to Peclet number (Pe) given by $Pe=\frac{{t}_{d}}{{t}_{e}}=\frac{{R}_{i}^{2}/{D}_{a}}{{t}_{e}}\propto \frac{1}{{L}_{c}}$ since *t*_*e*_ ∝ *L*_*c*_ where t_d_: time for diffusion, D_a_: diffusion coefficient of the particles in suspension (from Stokes-Einstein equation). Thus, Pe can be used to present a universal regime map to demarcate various final morphologies (Fig. 6). As can be seen from Fig. 6, there is a monotonic decrease in Pe with L_c_ and *φ*_*o*_. We also observe that for low Pe (*Pe* < 3 or high confinement say *L*_*c*_ = 20 *mm*) (Fig. 6), diffusion is comparable to the advection process (significant decrease in ${J}_{exit}^{^{\prime} }$) thereby limiting the migration of particles to the droplet periphery. Hence, the growth of any distinct thin shell is inhibited (Figs 2f,g and 4f) resulting in a NB-D or NB-T structures depending on φ_o_. NB-D cases do not buckle since ${P}_{cap}\propto J\propto \frac{1}{{L}_{c}} < {P}_{cr}$. In contrast, for *Pe* > 3 (low to moderate confinement), as the PLR is reduced (moving along the iso-confinement lines (Fig. 6)), the transition is from *B* & R → *B* → *NB* − *T* for L_c_ = 5–7 mm, *B* → *NB* − *T* for L_c_ = 10 mm and *B* → *NB* − *D* → *NB* − *T* for L_c_ = 15 mm. This is in contrast to unconfined case (L_c_ = 0 mm) where the morpho-dynamic transition is from *B* & R → *NB* − *T*. It is seen that rupturing is observed only for high Pe (i.e. low confinements). This is in corroboration with the fact that stretching ${\epsilon }\propto 1/{L}_{c}^{2}\propto {(Pe)}^{2}$.

**Figure 7:**
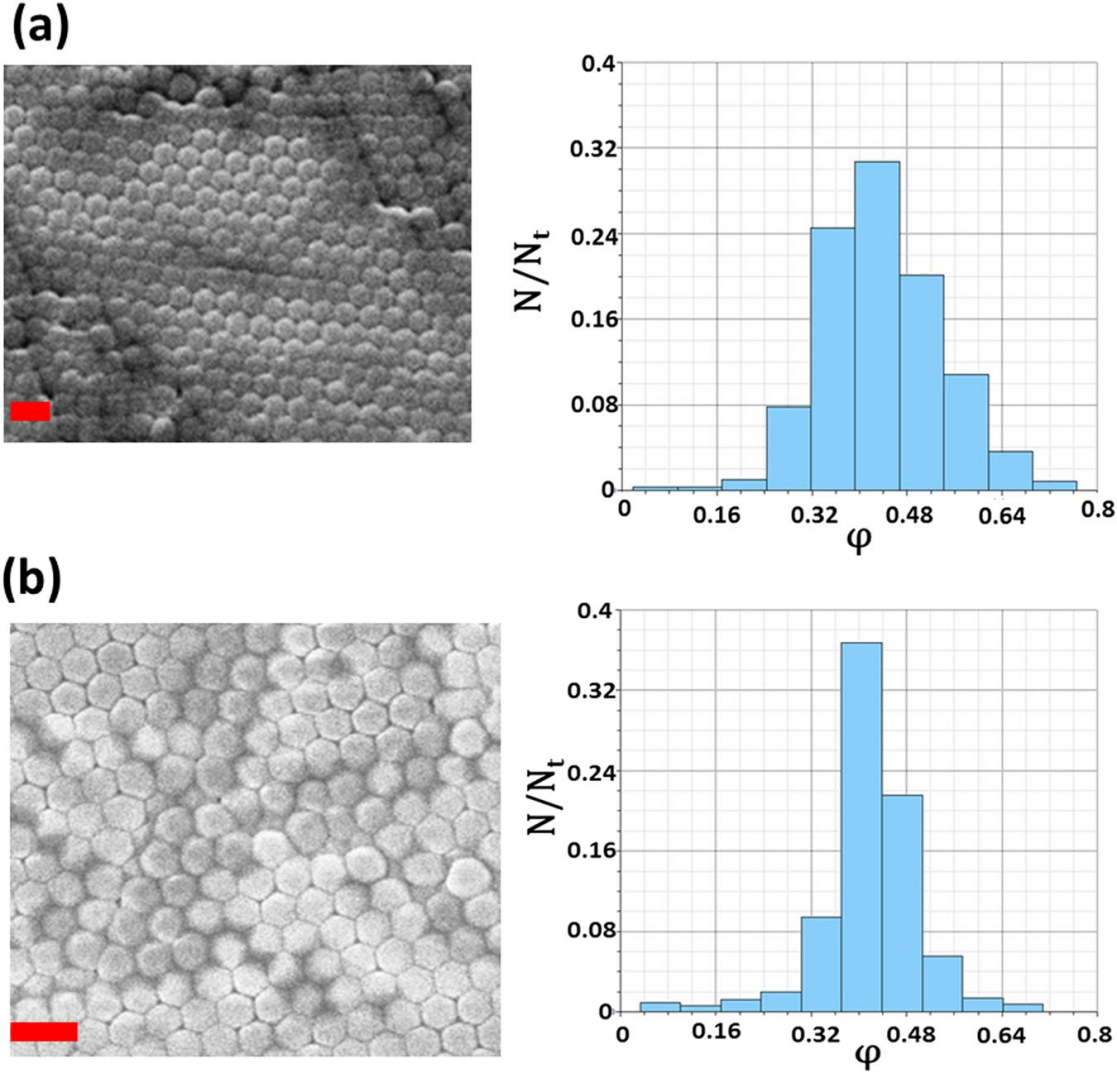
Particle arrangement and defects. SEM micrographs and histograms indicating the void fractions (φ) for (**a**) unconfined (**b**) confined droplet. Here each particle (N) is considered as a bin and void fraction is calculated for each bin. N_t_ is the total number of particles. Scale bar equals 400 nm. 200 nm particles are used to study the effect of confinement on particle arrangement.

As explained earlier, confining an evaporating droplet changes the macrostructure of the final precipitate. However, confinement can also affect the droplet morphology at the nanoscale i.e. it can modify the nanoparticles arrangement during evaporation induced self-assembly. To further investigate this, we used a 1 wt.% dispersion of polystyrene particles (Diameter ~200 nm) and repeated the experiments for unconfined and 20 mm channel confined cases. 200 nm particles are chosen as they have spectral wavelength in the visible region and are easy to analyze under SEM compared to 22 nm particles. Figure 7 shows SEM images of the close packed structures formed due to self-assembly for the two scenarios along with the histograms indicating the void fraction (φ) distributions. It can be seen from Fig. 7 that even though the peak values (~0.43) are close for the two cases, confined droplet (φ = 0.3–0.6) has a narrower range of void fractions than the unconfined scenario (φ = 0.2–0.7). The change in the distribution band is due to significant decrease in evaporation rate for confined cases resulting in more uniform packing of particles. In general, the unconfined droplets also exhibit comparatively larger void fractions (Fig. 7). This may cause a shift in reflectance wavelength. As reported in literature^25–27^, this can in turn affect the photonic band gaps as desired in photonic crystals. Moreover, packing fraction value (*φ*_*p*_ = 1 − *φ* = 0.57) from Fig. 7b is also in close correspondence with the packing fraction value of 0.56 obtained from loose random packing assumption.

## Discussion

To summarize, we have captured the effects of fluidic confinement on the morpho-dynamics of an evaporating nano-colloidal droplet. Wall effects induced by the confining boundaries result in vapor entrapment, which introduce an anisotropic flow field in the azimuthal direction. We provide a universal mapping between the flow velocity (scaling inversely with the channel length) and the evaporation rate. The alteration in the flow field due to confinement leads to preferential particle deposition, modifying the buckling behavior in a dramatic manner, with a possibility of complete suppression of the triggered instabilities beyond a critical channel length. In addition to the buckling regimes observed in unconfined droplets obtained by simply varying the initial particle concentration, the present work, thus, demonstrates a more complex regime map (based on dimensionless number) comprising of an additional tunable factor, i.e. the channel length. These findings are likely to have significant practical implications in the design of photonic crystal devices. Other potential applications include spray drying in pharmaceuticals and food industry, surface patterning^28, 29^, and fabrication of DNA chips^30^.

## Methods

### Experimental Methodology

A 1.5 µl deionized water droplet loaded with silica nanoparticles (particle diameter ~22 nm, Ludox TM40, Sigma Aldrich) at various concentrations (*φ*_*o*_ = 3 wt.% to 40 wt.%) is deployed inside a PDMS mini channel (2 mm width × 1.4 mm height; Fig. 1a). Droplets exhibit an initial apparent contact angle (CA), *θ*_*o*_ ≈ 110° ± 2° with contact radius *R*_*c*_ = 0.7 *mm*. Ambient conditions are maintained at 25 °C and 40% relative humidity. Images of the evaporating droplet (side view) are captured using a DSLR camera (Nikon D7200). Simultaneously, top view is acquired using a microscope with 5X objective. The acquired images are then processed to obtain temporally varying contact radius and contact angles over droplet lifetime. Macro and nanostructures of the final precipitates are captured using Scanning Electron Microscope (Zeiss Ultra 55). For visualizing flow field, 860nm rhodamine coated polystyrene particles are used. Images are captured (at $h/{h}_{i}=0.5\,$ where *h*_*i*_ is the initial droplet height) at 1 fps and processed using DAVIS-7.2 software. Experiments are repeated for *L*_*c*_ = 5 *mm* to 20 *mm* for same W and H (Fig. 1a). Confinement ratio (${V}_{c}/{V}_{D}\approx 2.8\,$ where *V*_*c*_ = *A*_*c*_ × 2*R*_*i*_, *A*_*c*_ is the cross-sectional area of the channel, *R*_*i*_ is the meridional radius and *V*_*D*_ is the initial droplet volume) is maintained constant across all the runs. IR thermograph is acquired using FLIR SC5200 camera. It is to be noted that for the present work, our aim is to study the buckling dynamics of an evaporating droplet under maximum confinement. Towards that, 1.5 µl was found to be the maximum volume which could be deployed inside the chosen channel geometry without touching the walls and still maintaining the maximum confinement (minimum free volume for a given channel length).

## Electronic supplementary material

Supplementary materials

Particle deposition for confined droplet
