# Supplementary material for: Confinement suppresses instabilities in particle-laden droplets
Source: Sci Rep. 2017 Aug 9;7:7708. doi: 10.1038/s41598-017-08126-3 (PMC5550431; doi:10.1038/s41598-017-08126-3)
Supplement: Supplementary file 1 — Supplementary materials [file 41598_2017_8126_MOESM1_ESM.pdf]

Supplementary Material

Confinement suppresses instabilities in particle-laden droplets

Lalit Bansal[1], Saptarshi Basu[1*], Suman Chakraborty[2*]

[1]Department of Mechanical Engineering,

Indian Institute of Science, Bangalore 560012, India

[2]Department of Mechanical Engineering,

Indian Institute of Technology Kharagpur, Kharagpur – 721302, India

*Corresponding author email: sbasu@mecheng.iisc.ernet.in; suman@mech.iitkgp.ernet.in

Experimental observations

Regression of contact radius

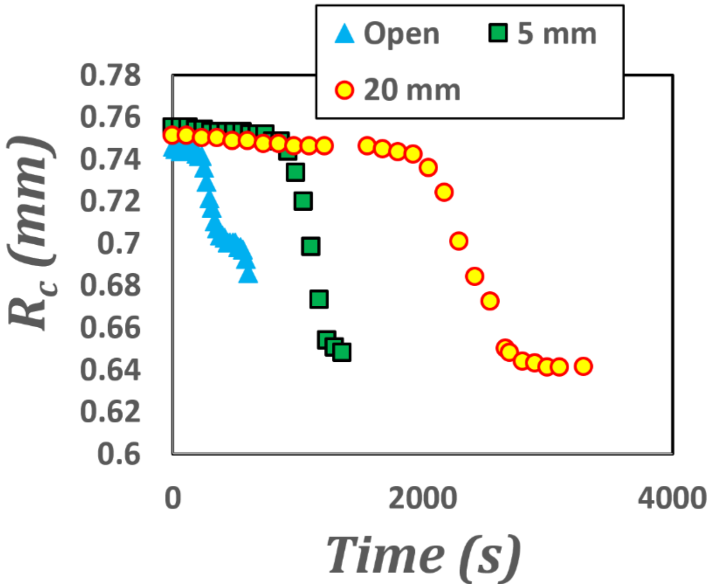

Fig. S1. Temporal variation of the contact radius for different configurations. Initially, there is CCR mode and then there is a sudden decrease in the radius. This decrease may be attributed to shrinkage of the droplet during solvent evaporation through the pores towards the end of its lifetime.

IR thermograph

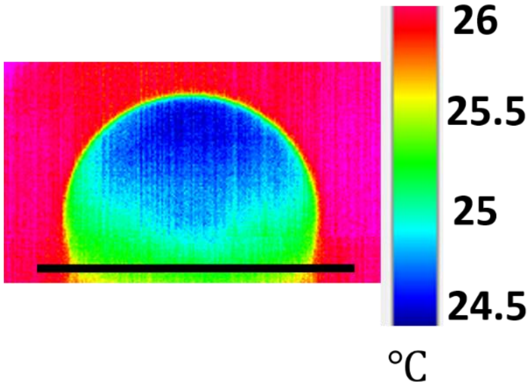

Fig. S2. IR thermograph of the unconfined droplet. The upper half of the droplet is comparatively cooler than the lower half due to higher evaporation rate (hence higher evaporative cooling). This creates a density gradient between the two halves resulting in a recirculating toroidal flow inside the droplet (Fig. 1d).

Calculation of altered evaporation flux

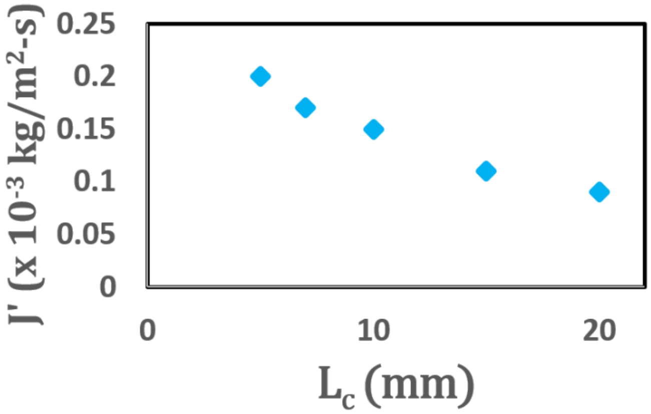

Figure S3. Variation in increased evaporation flux with channel length (L_c). To calculate the extent of accumulation of entrapped vapor inside the channel, we compare the diffusion from droplet to channel with that from channel to ambient[1] i.e.

$$\frac{2\pi DMR_c f(\theta)(c_s - c'_\infty)}{\rho} = \frac{DMA_{ch}(c'_\infty - c_\infty)}{\rho k' L_c}$$

Rearranging we have $k' = \frac{A_{ch}(c'_\infty - c_\infty)}{2\pi R_c f(\theta)(c_s - c'_\infty)L_a}$

Where k' is a factor which is a function of the channel length, A_{ch} is the channel area, c'_∞ is the increased channel vapor concentration, c_∞ is the ambient vapor concentration, R_c is the contact radius, $f(\theta) = (0.00008957 + 0.633\theta + 0.116\theta^2 - 0.08878\theta^3 + 0.01033\theta^4)/sin\theta$ [ref. 2], for $\theta > 10°$, θ is the contact angle and c_s is the saturated water vapor concentration.

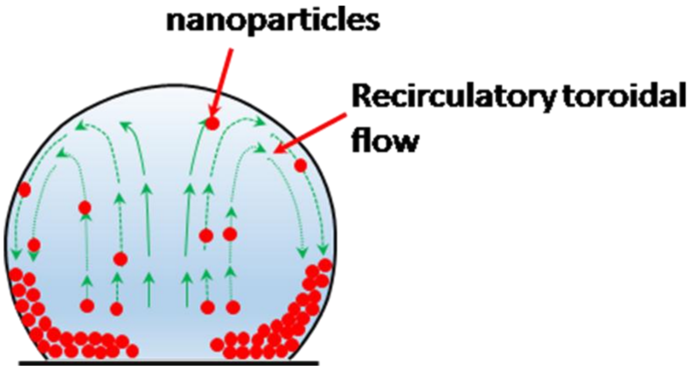

Fig. S4. Schematic showing the flow field inside an unconfined droplet transporting particles to the droplet contact line.

Calculation of shell thickness and buckling probability

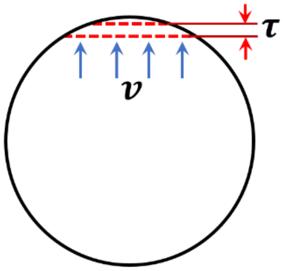

Fig. S5. Schematic representation of the evaporating droplet (top view). The dotted lines represent the control volume of thickness τ. Blue arrows represent the particle transport inside the control volume.

To theoretically estimate the instantaneous shell thickness, let us consider a control volume (CV) of thickness τ as shown in Fig. S5. Particles inside this CV are transported by the solvent and as the solvent evaporates, particles get deposited at the droplet periphery. Thus, mass conservation entails that the rate of change of mass inside the CV is directly correlated to the solvent (suspended with particles) flow, thus we have,

Rate of change of particles mass = Inflow of particles with the solvent

$$\varphi_p \rho \frac{dV}{dt} = \varphi_o \rho v_c dA \quad (1)$$

where φ_p is the particle packing fraction, ρ is the density, dV is the volume of CV, φ_o is the initial particle concentration, v is the flow velocity and dA is the cross-sectional area of the CV.

Eqn. 1 can be modified as

$$\varphi_p \frac{d\tau}{dt} = \varphi_o v_c$$

$$d\tau = \frac{\varphi_o}{\varphi_p} v_c dt \quad (2)$$

Subscript 'c' stands for confined droplet. Using the condition, at $t = t_i$, shell thickness, $\tau = 0$, where t_i is the time instant at which particles begin to aggregate on the periphery, thus Eqn. 2 becomes,

$$\tau = \frac{\varphi_o}{\varphi_p} v_c (t_i - t)$$

As the shell grows in thickness, it may buckle if the capillary pressure exerted by the evaporation through the nanopores exceeds the critical pressure required for buckling.

Morphology of the precipitates (SEM images)

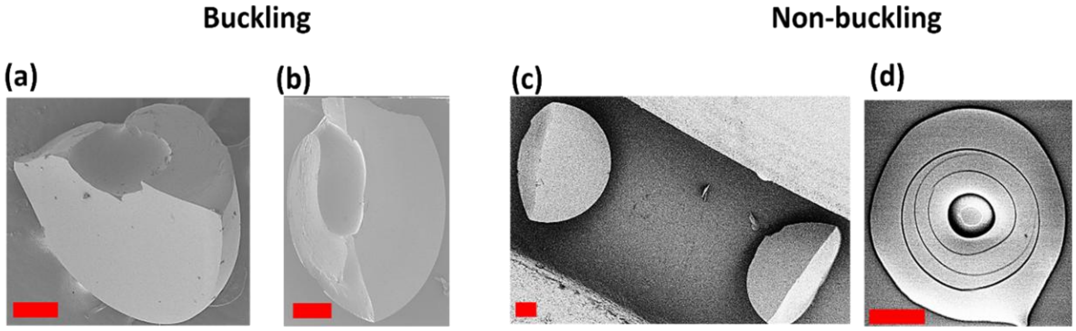

Fig. S6. SEM micrographs of the final precipitates. (a,d) L_c=5 mm, (b) L_c=7 mm, (c) L_c=20 mm. (a), (b) and (c) correspond to initial particle concentration of 40 wt.% while (d) corresponds to 5 wt.%. (a) and (b) show Buckling-Rupturing regime with decreasing cavity volume as the channel length is increased, (c) shows solid structure without any cavity. In this case, due to low evaporation flux, a dome shaped structure is obtained (d) thin disc is formed due to insufficient number of particles required for shell formation. Scale bar equals 200 µm.

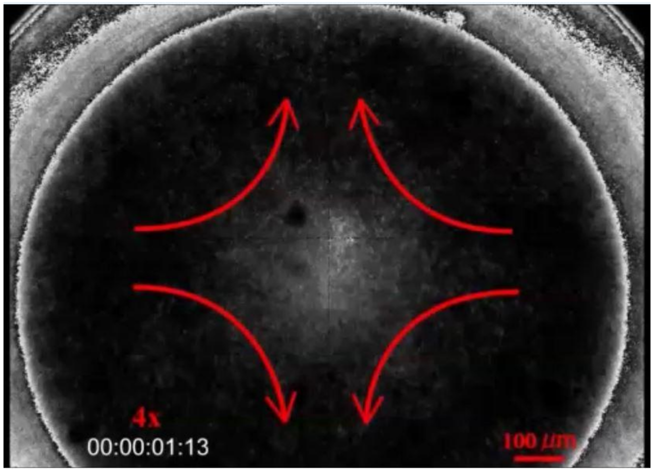

Movie M1: Particle deposition for confined droplet.

References

1. L. Bansal, S. Chakraborty, and S. Basu. Soft Matter **13**, 969 (2017).
2. Y. O. Popov. Physical Review E **71**, 036313 (2005).
